# Prognostic nutritional index: A potential biomarker for predicting the prognosis of decompensated liver cirrhosis

**DOI:** 10.3389/fnut.2022.1092059

**Published:** 2023-01-06

**Authors:** Yanan Xie, Chiyi He, Wei Wang

**Affiliations:** Department of Gastroenterology, Yijishan Hospital of Wannan Medical College, Wuhu, Anhui, China

**Keywords:** decompensated liver cirrhosis, prognostic nutritional index, prognostic factor, mortality, independent predictor, validation

## Abstract

**Background:**

Prognostic nutritional index (PNI) is an independent predictor of the prognosis of various diseases. However, the prognosis value of PNI in patients with decompensated liver cirrhosis (DLC) remains unknown. The study aimed to investigate the prognostic significance of PNI in patients with DLC.

**Methods:**

A total of 214 eligible patients were enrolled in the study’s development cohort between January 2018 and March 2021. The clinical primary study endpoints were mortality at 3 and 6 months. Receiver operating characteristic (ROC) curve analysis was used to assess the PNI’s prediction accuracy, and Youden’s index was utilized to determine the PNI’s optimal cut-off value. Moreover, based on the optimal cut-off value, patients were categorized into high and low PNI groups. Multivariate logistic regression analysis was used to determine independent risk factors for mortality, while the relationship between PNI and the risk of death was identified and demonstrated using restricted cubic splines (RCS). A validation cohort of 139 patients was to verify the predictive power of the PNI.

**Results:**

In the development cohort, the mortality rate at 3 and 6 months were 10.3% (22) and 14.0% (30), respectively. The PNI had comparable predictive power with the MELD score at all follow-up endpoints. Decreased PNI was an independent predictor of adverse prognosis at all follow-up endpoints. The RCS revealed a linear correlation between PNI and the risk of death. We confirmed that lower PNI was an independent predictor of poor prognosis in the validation cohort.

**Conclusion:**

The findings showed that lower PNI is an independent factor of poor outcomes and might be utilized as a potentially promising prognostic predictor in patients with DLC.

## Introduction

Liver cirrhosis possesses a significant global morbidity and mortality rate, resulting in a million deaths annually, and is the 11th leading cause of death globally (1, 2). As compensated liver cirrhosis is difficult to identify, most patients are diagnosed with decompensated liver cirrhosis (DLC) in the hospital due to numerous complications (3). DLC has an unfavorable prognosis, with a median survival time of approximately 2 years, placing a heavy financial burden on healthcare (4). Despite the availability of several therapies, the mortality rate remains high for patients with DLC (5, 6). As a result, there is a need for a practical and simple predictor to evaluate the risk of death in patients with DLC in order to improve clinical management and subsequently reduce mortality.

Prognostic Nutritional Index (PNI) is a simple and objective index of inflammatory and nutrition status derived from serum albumin (ALB) and lymphocyte counts. Recently, PNI has been reported to be an independent prognostic predictor for patients with cancer, stroke, heart disease, chronic kidney disease, acute exacerbation of the chronic obstructive pulmonary disease, sepsis, COVID-19, and autoimmune disease (7–17). However, no research has been conducted to examine the relationship between PNI and the prognosis of patients with DLC. Several studies have shown that systemic inflammatory response and malnutrition are associated with poor prognosis in patients with liver cirrhosis (18–24). Hence, it seems reasonable to hypothesize that there may be a significant correlation between PNI and the risk of death in patients with DLC when PNI is used as an indicator of inflammatory and nutrition status. The current study aimed to evaluate the prognostic role of PNI in patients with DLC.

## Materials and methods

### Patient population

Between January 2018 and March 2021, we recruited patients with DLC who were admitted to the Department of Gastroenterology, Yijishan Hospital of Wannan Medical College as the development cohort of the study. We enrolled patients with DLC attending the Yijishan Hospital of Wannan Medical College between April 2021 and February 2022 as the validation cohort. DLC was defined as biochemical, clinical, endoscopic manifestations, imaging signs, and complications of ascites, gastrointestinal bleeding, hepatorenal syndrome, or hepatic encephalopathy (25). Reasons for exclusion were: (1) non-first admission, (2) malignant diseases, (3) cardio-cerebrovascular disease, (4) autoimmune diseases, (5) hyperpyrexia, (6) primary kidney disease, (7) incomplete data, and (8) loss to follow-up. The flow chart of the patient selection process is provided in Supplementary Figure 1.

### Study variables and outcomes

On admission, variables including sex, age, cause of liver cirrhosis, modes of decompensation, and laboratory variables were collected (Table 1). PNI was calculated as serum albumin (ALB) concentration (g/L) + 5 × total lymphocyte count (10^9^/L) (16). The Model for End-Stage Liver Disease (MELD) score was utilized to evaluate the severity and prognosis of liver disease (26). The mortality rate at 3 and 6 months was assessed using medical records or direct telephone conversations with patients or their relatives.

**TABLE 1:** Clinical and laboratory characteristics of the decompensated liver cirrhosis patients in the development and validation cohort at the 3-month, and 6-month follow-ups.

Variables	Development cohort							Validation cohort						
	All patients (*n* = 214)	3 months			6 months			All patients (*n* = 139)	3 months			6 months		
		Survivors (*n* = 192)	Non-survivors (*n* = 22)	*P*	Survivors (*n* = 184)	Non-survivors (*n* = 30)	*P*		Survivors (*n* = 123)	Non-survivors (*n* = 16)	*P*	Survivors (*n* = 115)	Non-survivors (*n* = 24)	*P*
Gender (*n*, %)				0.161			0.054				0.830			0.859
Male	127 (59.3%)	117 (60.9%)	10 (45.5%)		114 (62.0%)	13 (43.3%)		73 (52.5%)	65 (52.8%)	8 (50.0%)		60 (52.2%)	13 (54.2%)	
Female	87 (40.7%)	75 (39.1%)	12 (54.5%)		70 (38.0%)	17 (56.7%)		66 (47.5%)	58 (47.2%)	8 (50.0%)		55 (47.8%)	11 (45.8%)	
Age (years)	61.4 ± 12.9	61.1 ± 13.0	64.6 ± 11.7	0.228	60.8 ± 12.7	65.1 ± 13.7	0.094	61.3 ± 12.5	60.3 ± 12.5	68.9 ± 10.1	0.009	60.3 ± 12.8	66.3 ± 10.1	0.016
WBC (10^9^/L)	3.8 (2.6–5.4)	3.6 (2.5–5.1)	5.1 (3.9–6.4)	0.004	3.6 (2.5–5.1)	4.5 (3.6–6.6)	0.005	3.8 (2.8–5.9)	3.8 (2.8–5.8)	5.1 (2.7–10.0)	0.138	3.8 (2.8–5.8)	4.6 (2.8–7.5)	0.182
LYM (10^9^/L)	0.8 (0.6–1.2)	0.8 (0.6–1.2)	0.9 (0.5–1.2)	0.893	0.8 (0.6–1.2)	0.8 (0.5–1.2)	0.940	1.0 (0.6–1.4)	1.0 (0.6–1.4)	0.8 (0.5–1.2)	0.128	1.0 (0.6–1.5)	0.8 (0.5–1.2)	0.066
HGB (g/L)	96.3 ± 25.4	96.4 ± 24.7	95.9 ± 31.1	0.932	96.3 ± 25.1	96.6 ± 27.3	0.944	93.7 ± 27.7	95.8 ± 27.3	77.5 ± 25.8	0.012	96.7 ± 27.6	79.5 ± 23.6	0.005
PLT (10^9^/L)	61.5 (44.0–98.5)	58.0 (43.3–94.0)	92.5 (62.8–152.3)	0.007	57.5 (43.3–90.8)	98.5 (62.8–130.8)	0.004	73.0 (49.0–101.0)	71.0 (48.0–101.0)	88.0 (63.0–115.3)	0.151	73.0 (48.0–102.0)	69.0 (49.5–96.8)	0.789
ALB (g/L)	29.6 ± 6.2	30.0 ± 6.2	26.0 ± 4.9	0.004	30.2 ± 6.1	25.8 ± 5.0	<0.001	29.7 ± 5.6	30.2 ± 5.5	25.6 ± 4.7	0.002	30.5 ± 5.6	25.8 ± 4.1	<0.001
TBIL (μmol/L)	24.8 (16.6–37.5)	24.4 (16.8–36.6)	34.1 (16.5–56.2)	0.170	24.5 (17.4–36.8)	27.1 (15.3–51.5)	0.669	27.8 (17.7–45.6)	27.2 (16.9–45.4)	36.8 (22.0–75.9)	0.160	26.9 (16.9–44.4)	36.1 (22.0–61.9)	0.087
ALT (U/L)	25.0 (16.0–41.0)	25.0 (17.0–41.0)	23.5 (13.3–41.5)	0.465	25.0 (17.0–41.8)	23.0 (14.8–34.3)	0.241	24.0 (16.0–41.0)	25.0 (19.0–45.0)	16.0 (9.3–25.3)	0.002	25.0 (19.0–45.0)	18.0 (10.5–28.3)	0.008
AST (U/L)	34.0 (23.0–58.0)	33.0 (22.3–58.0)	43.0 (26.8–59.8)	0.422	34.0 (22.3–58.0)	32.5 (26.0–55.0)	0.994	35.0 (23.0–60.0)	37.0 (24.0–61.0)	26.5 (18.3–37.8)	0.065	37.0 (24.0–61.0)	28.5 (18.3–44.0)	0.139
GGT (U/L)	54.5 (23.0–145.3)	55.5 (23.5–136.3)	46.5 (18.8–200.3)	0.815	55.5 (25.0–132.5)	47.0 (18.8–214.8)	0.757	44.0 (21.0–98.0)	48.0 (24.0–110.0)	21.0 (12.3–59.3)	0.017	49.0 (24.0–110.0)	21.0 (14.0–59.3)	0.015
BUN (mmol/L)	5.4 (4.2–8.0)	5.4 (4.2–7.7)	8.1 (4.3–11.9)	0.084	5.4 (4.2–7.7)	6.5 (4.6–11.9)	0.131	6.3 (4.7–9.6)	6.0 (4.5–8.9)	10.1 (7.0–13.4)	0.001	5.9 (4.3–8.9)	9.5 (6.1–11.7)	0.001
Cr (μmol/L)	67.3 (56.0–85.3)	67.3 (55.9–84.4)	69.7 (54.2–120.4)	0.383	67.3 (56.1–84.3)	68.1 (54.4–104.7)	0.532	59.2 (47.4–81.0)	58.7 (47.0–76.5)	75.6 (55.1–133.8)	0.021	58.7 (46.7–76.4)	69.9 (54.2–117.4)	0.032
PT (S)	14.6 (13.3–16.2)	14.4 (13.2–16.2)	15.7 (14.4–17.2)	0.050	14.4 (13.3–16.1)	15.7 (13.1–19.2)	0.080	15.1 (13.8–16.6)	14.9 (13.7–16.5)	15.9 (14.7–17.9)	0.016	14.8 (13.4–16.2)	15.9 (15.1–18.9)	0.002
PNI	34.6 ± 6.7	35.0 ± 6.6	31.1 ± 7.0	0.01	35.1 ± 6.6	31.0 ± 6.7	0.002	34.2 (30.6–39.8)	35.2 (31.3–40.2)	30.2 (26.6–33.6)	<0.001	35.8 (31.6–40.5)	30.4 (27.8–32.9)	<0.001
MELD score	11.2 (9.1–13.6)	11.2 (9.0–13.2)	14.1 (10.6–17.9)	0.005	11.2 (9.0–13.0)	13.6 (10.0–17.3)	0.017	11.5 (9.0–14.9)	10.9 (9.0–14.3)	15.4 (12.1–21.1)	0.002	10.7 (9.0–14.3)	14.2 (11.7–20.5)	0.001
**Etiology (*n*, %)**														
HBV	134 (62.6%)	119 (62.0%)	15 (68.2%)		112 (60.9%)	22 (73.3%)		84 (60.4%)	76 (61.8%)	8 (50.0%)		71 (61.7%)	13 (54.2%)	
HCV	7 (3.3%)	7 (3.6%)	0		7 (3.8%)	0		7 (5.0%)	6 (4.9%)	1 (6.3%)		6 (5.2%)	1 (4.2%)	
Alcoholism	5 (2.3%)	5 (2.6%)	0		5 (2.7%)	0		5 (3.6%)	5 (4.1%)	0		4 (3.5%)	1 (4.2%)	
Others	68 (31.8%)	61 (31.8%)	7 (31.8%)		60 (32.6%)	8 (26.7%)		43 (30.9%)	36 (29.3%)	7 (43.8%)		34 (29.6%)	9 (37.5%)	
**Modes of decompensation (*n*, %)**														
Ascites	181 (84.6%)	162 (84.4%)	19 (86.4%)		155 (84.2%)	26 (86.7%)		120 (86.3%)	105 (85.4%)	15 (93.8%)		99 (86.1%)	21 (87.5%)	
Variceal bleeding	69 (32.2%)	64 (33.3%)	5 (22.7%)		63 (34.2%)	6 (20.0%)		44 (31.7%)	39 (31.7%)	5 (31.3%)		36 (31.3%)	8 (33.3%)	
HE	9 (4.2%)	5 (2.6%)	4 (18.2%)		5 (2.7%)	4 (13.3%)		4 (2.9%)	1 (0.8%)	3 (18.8%)		1 (0.9%)	3 (12.5%)	
HRS	5 (2.3%)	1 (0.5%)	4 (18.2%)		0	5 (16.7%)		3 (2.2%)	0	3 (18.8%)		0	3 (12.5%)	

Data are expressed as number, mean ± standard deviation, median (25th–75th percentiles), or frequency [percentage (%)]. WBC, white blood cell; LYM, lymphocyte; HGB, hemoglobin; PLT, platelet; ALB, albumin; TBIL, total bilirubin; ALT, alanine aminotransferase; AST, aspartate aminotransferase; GGT, γ–glutamyl transpeptidase; BUN, blood urea nitrogen; Cr, creatinine; PT, prothrombin time; PNI, prognostic nutritional index; MELD, Model for End–Stage Liver Disease; HBV, hepatitis B virus; HCV, hepatitis C virus; HE, hepatic encephalopathy; HRS, hepatorenal syndrome.

### Statistical analysis

Categorical data were reported as frequency and percentage, while continuous variables were expressed as means ± standard deviation or medians (25th–75th percentiles). The independent sample *t*-test (normally distributed data) or the Mann-Whitney *U*-test (non-normally distributed data) were used to examine the differences in continuous variables. Categorical data were assessed by the chi-square test (27). Associations between MELD score and PNI were analyzed using Spearman’s analysis (28). Multivariate logistic regression analysis was used to determine independent predictors of mortality in patients with DLC. The degree of multicollinearity among the variables was measured by calculating the variance inflation factor (VIF) values, and VIF > 10 was considered to have multicollinearity (29). The diagnostic accuracy of PNI was assessed by analyzing the area under the receiver operating characteristic (ROC) curve (30), and the PNI’s optimal cutoff value was determined using Youden’s index (31). The values of the area under the ROC curve (AUC) were compared using the DeLong test (32). The patients were then categorized into high and low groups based on the optimal cut-off value. The Kaplan-Meier analysis with log-rank test was used to estimate survival between high and low groups. Furthermore, based on multivariate analysis, restricted cubic spline (RCS) was applied to assess the non-linear association between the PNI and the risk of death (33). The number of knots between three and five was chosen based on the minimum value for the Akaike information criterion to obtain the best fit and avoid overfitting in the main splines (34). Two-side *P*-value < 0.05 was considered statistically significant. The SPSS (version 25.0), R (version 4.0.2), and MedCalc (Version 15.2) were used to perform the statistical analyses in the study.

## Results

### Study population

A total of 353 patients with DLC who met the inclusion criteria were recruited for the study. In the development cohort, ascites (84.6%) were found to be the most common type of decompensation, followed by variceal bleeding (32.2%), hepatic encephalopathy (4.2%), and hepatorenal syndrome (2.3%). Cirrhosis was caused by chronic hepatitis B virus in most cases, and the patients’ average age was 61.4 ± 12.9 years. The mortality rate at 3 and 6 months were 10.3 and 14.0%, respectively. The baseline characteristics of patients in the development and validation cohort are shown in Table 1 and Supplementary Figure 2.

### Clinical and laboratory characteristics between non-survivors and survivors

In the development cohort, the PNI and ALB of the survivor were significantly higher than the non-survivor, while the white blood cell (WBC), platelet (PLT), and MELD scores of the survivor were considerably lower than the non-survivor at any phase of the follow-up (*P* < 0.05). At all follow-up endpoints, no significant differences were found between the two groups in lymphocyte (LYM), hemoglobin (HGB), total bilirubin (TBIL), alanine aminotransferase (ALT), aspartate aminotransferase (AST), γ–glutamyl transpeptidase (GGT), blood urea nitrogen (BUN), creatinine (Cr), and gender. The clinical and laboratory characteristics between non-survivors and survivors in the development and validation cohort are shown in Table 1.

### Clinical and laboratory characteristics in high and low PNI groups

**FIGURE 1:**
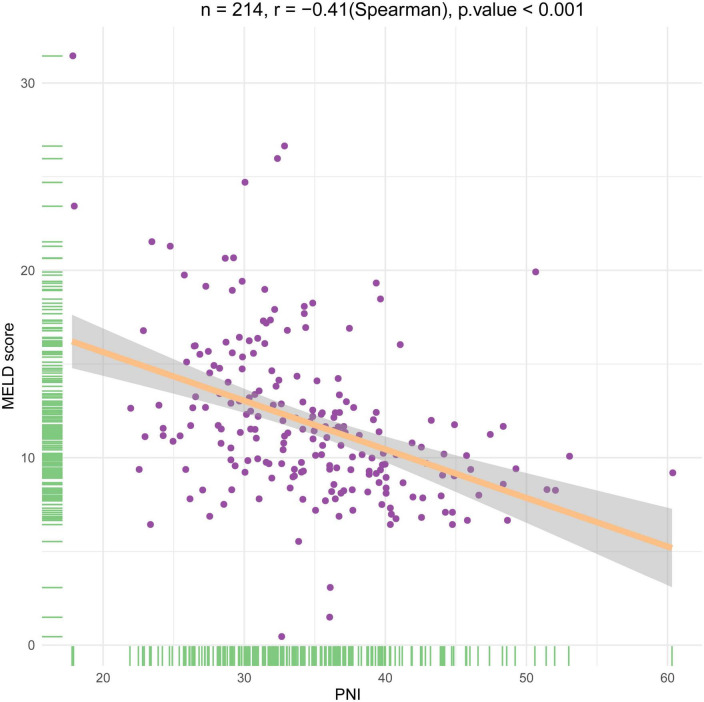
Scatter graphs illustrating the association between the prognostic nutritional index and the MELD score.

In the development cohort, patients were categorized into two groups based on Youden’s index, with cut-off values of 35.47 at 3, and 6 months. Patients in the low PNI group were significantly associated with increased mortality, TBIL, PT, and MELD score, and decreased LYM, ALB, and HGB compared to those of the high PNI group at all follow-up endpoints (Table 2). Furthermore, a negative correlation was found between PNI and MELD score (*r* =–0.41, *P* < 0.001) (Figure 1). In addition, the clinical and laboratory characteristics of patients in the high and low PNI groups in the validation cohort are shown in Table 2.

**TABLE 2:** Comparison of clinical and laboratory characteristics between low and high PNI groups in the development and validation cohort in patients with decompensated liver cirrhosis.

Variables	Development cohort						Validation cohort					
	3 months			6 months			3 months			6 months		
	PNI < 35.47 (*n* = 122)	PNI > 35.47 (*n* = 92)	*P*	PNI < 35.47 (*n* = 122)	PNI > 35.47 (*n* = 92)	*P*	PNI < 35.47 (*n* = 79)	PNI > 35.47 (*n* = 60)	*P*	PNI < 35.47 (*n* = 79)	PNI > 35.47 (*n* = 60)	*P*
Gender (*n*, %)			0.196			0.196			0.861			0.861
Male	77 (63.1%)	50 (54.3%)		77 (63.1%)	50 (54.3%)		42 (53.2%)	31 (51.7%)		42 (53.2%)	31 (51.7%)	
Female	45 (36.9%)	42 (45.7%)		45 (36.9%)	42 (45.7%)		37 (46.8%)	29 (48.3%)		37 (46.8%)	29 (48.3%)	
Age (years)	61.0 ± 13.5	62.0 ± 12.1	0.578	61.0 ± 13.5	62.0 ± 12.1	0.578	62.6 ± 12.4	59.6 ± 12.7	0.164	62.6 ± 12.4	59.6 ± 12.7	0.164
WBC (10^9^/L)	3.7 (2.4–5.5)	3.9 (2.7–5.3)	0.557	3.7 (2.4–5.5)	3.9 (2.7–5.3)	0.557	3.7 (2.4–5.8)	4.1 (3.1–6.2)	0.166	3.7 (2.4–5.8)	4.1 (3.1–6.2)	0.166
HGB (g/L)	91.7 ± 24.3	102.4 ± 25.5	0.002	91.7 ± 24.3	102.4 ± 25.5	0.002	87.9 ± 23.6	101.3 ± 30.8	0.004	87.9 ± 23.6	101.3 ± 30.8	0.004
PLT (10^9^/L)	59.0 (41.0–92.5)	65.5 (48.3–108.3)	0.094	59.0 (41.0–92.5)	65.5 (48.3–108.3)	0.094	63.0 (44.0–98.0)	81.5 (52.0–116.8)	0.022	63.0 (44.0–98.0)	81.5 (52.0–116.8)	0.022
TBIL (μmol/L)	28.7 (18.1–48.3)	21.5 (15.0–33.4)	0.001	28.7 (18.1–48.3)	21.5 (15.0–33.4)	0.001	31.8 (19.8–54.8)	24.4 (15.0–37.8)	0.001	31.8 (19.8–54.8)	24.4 (15.0–37.8)	0.070
ALT (U/L)	25.0 (15.0–42.3)	25.0 (17.0–40.8)	0.917	25.0 (15.0–42.3)	25.0 (17.0–40.8)	0.917	23.0 (16.0–37.0)	24.0 (19.0–45.8)	0.279	23.0 (16.0–37.0)	24.0 (19.0–45.8)	0.279
AST (U/L)	37.0 (23.0–65.0)	31.0 (22.0–52.0)	0.094	37.0 (23.0–65.0)	31.0 (22.0–52.0)	0.094	37.0 (23.0–60.0)	34.5 (22.3–60.5)	0.975	37.0 (23.0–60.0)	34.5 (22.3–60.5)	0.975
GGT (U/L)	47.5 (19.8–155.3)	62.0 (28.5–140.8)	0.232	47.5 (19.8–155.3)	62.0 (28.5–140.8)	0.232	36.0 (18.0–83.0)	54.0 (24.5–150.8)	0.078	36.0 (18.0–83.0)	54.0 (24.5–150.8)	0.078
BUN (mmol/L)	6.0 (4.2–8.8)	5.2 (4.2–6.4)	0.060	6.0 (4.2–8.8)	5.2 (4.2–6.4)	0.060	6.9 (4.7–10.5)	5.8 (4.6–8.6)	0.044	6.9 (4.7–10.5)	5.8 (4.6–8.6)	0.044
Cr (umol/L)	69.8 (58.2–90.8)	64.6 (54.4–82.1)	0.061	69.8 (58.2–90.8)	64.6 (54.4–82.1)	0.061	60.7 (48.4–81.0)	57.4 (47.1–81.5)	0.230	60.7 (48.4–81.0)	57.4 (47.1–81.5)	0.230
PT (S)	15.5 (14.0–16.9)	13.7 (12.7–14.9)	<0.001	15.5 (14.0–16.9)	13.7 (12.7–14.9)	<0.001	15.6 (14.5–17.7)	14.2 (12.9–15.4)	< 0.001	15.6 (14.5–17.7)	14.2 (12.9–15.4)	<0.001
PNI	30.0 ± 3.7	40.6 ± 4.8	<0.001	30.0 ± 3.7	40.6 ± 4.8	<0.001	31.2 (27.9–33.0)	40.4 (37.4–43.8)	< 0.001	31.2 (27.9–33.0)	40.4 (37.4–43.8)	<0.001
MELD score	12.6 (10.2–16.0)	9.4 (8.1–11.4)	<0.001	12.6 (10.2–16.0)	9.4 (8.1–11.4)	<0.001	12.5 (10.0–16.3)	10.3 (8.3–12.6)	< 0.001	12.5 (10.0–16.3)	10.3 (8.3–12.6)	<0.001
Mortality (*n*, %)	20 (16.4%)	2 (2.2%)	0.001	27 (22.1%)	3 (3.3%)	<0.001	15 (19.0%)	1 (1.7%)	0.002	23 (29.1%)	1 (1.7%)	<0.001

Data are presented as number, mean ± standard deviation, median (25th–75th percentiles), or frequency [percentage (%)]. WBC, white blood cell; LYM, lymphocyte; HGB, hemoglobin; PLT, platelet; ALB, albumin; TBIL, total bilirubin; ALT, alanine aminotransferase; AST, aspartate aminotransferase; GGT, γ–glutamyl transpeptidase; BUN, blood urea nitrogen; Cr, creatinine; PT, prothrombin time; PNI, prognostic nutritional index; MELD, Model for End–Stage Liver Disease.

### Low PNI as an independent factor of poor prognosis in patients with DLC

**FIGURE 2:**
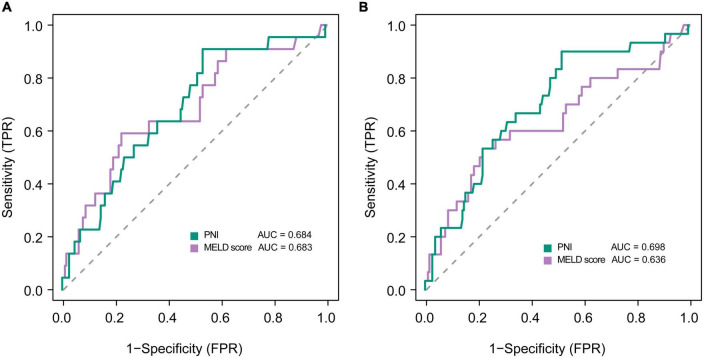
Receiver operating curves showing predictive accuracy of the MELD score and the PNI for mortality at **(A)** 3, and **(B)** 6 months in patients with decompensated liver cirrhosis.

Kaplan-Meier analyses showed that mortality was significantly higher in patients with low PNI group than that of in patients with high PNI group (Supplementary Figure 3). In multivariate logistic regression analysis, lower PNI was identified as an independent predictor of adverse outcomes in patients with DLC in the development cohort after adjusting for the effect of confounders on mortality at 3, and 6 months, respectively (Table 3). The ROC analysis demonstrated that the AUC values of PNI and MELD scores were comparable at 3 months (0.684 vs. 0.683). The AUC values of PNI were higher than the MELD score (0.698 vs. 0.636) at 6 months, but the difference was not statistically significant (Delong test *P*-value > 0.05) (Figure 2).

**TABLE 3:** Factors correlated with 3-month, and 6-month mortality in multivariate analyses in patients with decompensated liver cirrhosis in the development cohort and validation cohort.

Variables	Development cohort				Validation cohort			
	3 months OR (95% CI)	*P*	6 months OR (95% CI)	*P*	3 months OR (95% CI)	*P*	6 months OR (95% CI)	*P*
WBC (10^9^/L)	1.098 (0.935–1.280)	0.230	1.107 (0.957–1.279)	0.160	1.173 (0.951–1.469)	0.138	1.203 (0.994–1.486)	0.065
PLT (10^9^/L)	1.005 (1.000–1.011)	0.044	1.005 (1.000–1.010)	0.072	1.005 (0.990–1.020)	0.469	0.999 (0.983–1.012)	0.837
AST (U/L)	1.002 (0.998–1.005)	0.349			0.990 (0.970–1.004)	0.238		
BUN (mmol/L)	1.086 (0.981–1.212)	0.122			1.087 (0.961–1.218)	0.160		
PT (S)	1.057 (0.843–1.329)	0.628	1.045 (0.868–1.261)	0.639	0.963 (0.757–1.152)	0.670	1.120 (0.945–1.408)	0.256
MELD score	1.059 (0.905–1.237)	0.469	1.076 (0.950–1.213)	0.232	1.167 (0.977–1.411)	0.095	1.057 (0.917–1.214)	0.430
PNI		0.037		0.006		0.036		0.006
Low	Reference		Reference		Reference		Reference	
High	0.187 (0.028–0.756)		0.162 (0.036–0.530)		0.098 (0.005–0.592)		0.053 (0.003–0.283)	

WBC, white blood cell; PLT, platelet; AST, aspartate aminotransferase; BUN, blood urea nitrogen; PT, prothrombin time; MELD, Model for End-Stage Liver Disease; PNI, prognostic nutritional index; OR, odds ratio; CI, confidence interval. Age, sex, hemoglobin, WBC, PLT, AST, BUN, PT, PNI, MELD score, total bilirubin, alanine aminotransferase, γ–glutamyl transpeptidase, and creatinine were included in the univariate logistic regression analysis. Variables that did not have a significant effect on mortality in the univariate logistic regression analysis were not included in the multivariate logistic regression analysis.

### Linear relationship between the PNI and risk of death

**FIGURE 3:**
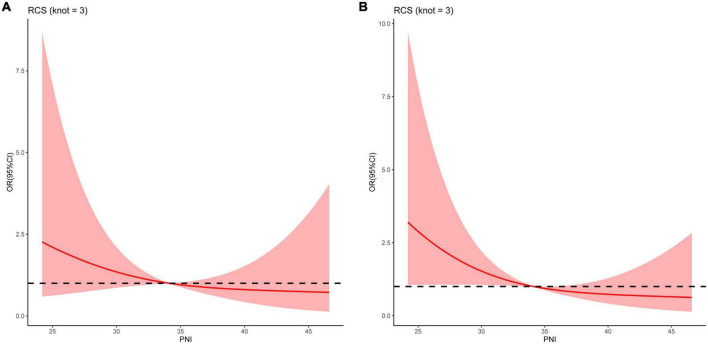
Restricted cubic spline curves for the relationships between the prognostic nutritional index and the risk of death at **(A)** 3, and **(B)** 6 months in patients with decompensated liver cirrhosis.

A linear association was observed between the PNI and the risk of death at all follow-up time points (all *P* for non-linearity > 0.05) (Figure 3). PNI was found to be negatively associated with the risk of death, indicating that the risk of death increased with the decrease in PNI.

### Verification of the predictive power of the PNI

In the validation cohort, we confirmed that the PNI was an independent predictor of 3- and 6-month mortality in patients with DLC (Table 3). In addition, the ROC analysis demonstrated that the PNI had a comparable predictive ability with the MELD score (All Delong test *P*-value > 0.05) (Supplementary Figure 4).

## Discussion

The findings demonstrated that lower PNI was an independent predictor for adverse outcomes at all follow-up endpoints and PNI had a potential predictive value for mortality in patients with DLC. Furthermore, a linear correlation between PNI and the risk of death was observed, indicating that mortality increased with the decrease in PNI. Currently, the MELD score is the most extensively used scoring system for stratifying disease severity and predicting mortality in advanced liver disease but requires complicated calculations that are inconvenient for clinical practice (26). In contrast, PNI is a straightforward, effective, and simple index that uses serum albumin level and total lymphocyte count (8). Our findings revealed a significant negative correlation between PNI and MELD scores, and indicated that PNI had comparable predictive power to MELD score at all follow-up endpoints. Previous research found some inflammatory and nutrition indicators, such as the albumin-bilirubin scores, the international normalized ratio-to-albumin ratio, the neutrophil-to-albumin ratio, and the neutrophil-to-lymphocyte ratio, were independent predictors of mortality in patients with liver cirrhosis (35–38). To our knowledge, this is the first study which identified that PNI could be used as an independent predictor for mortality in patients with DLC.

PNI is a simple index developed by Onodera et al., reflecting immune, inflammatory, and nutritional status (39). According to recent studies, PNI significantly correlates with adverse outcomes in various diseases (7–17). Zheng et al. reported that a lower PNI is an independent risk factor for higher mortality in patients with respectable esophageal squamous cell carcinoma (7). According to Chen et al., a lower PNI is independently correlated with increased cardiovascular disease death and overall mortality in patients with heart failure (11). Bodolea et al. found that a lower PNI is an independent predictor of higher mortality in patients with severe COVID-19 (17). Similarly, our results showed that a lower PNI is an independent predictor of poor prognosis in patients with DLC.

There are specific explanations as to why PNI can predict the prognosis in patients with DLC. Firstly, albumin can reflect systemic inflammation and nutritional status (40, 41). Serum albumin had a negative relationship with the intensity of the systemic inflammatory response (37, 38). It has been recognized to have a significant role in the pathogenesis of end-stage liver cirrhosis and is related to poor outcome (21–24). A recent study has also demonstrated that low serum albumin may be caused by a combination of hepatic reorganization of protein synthesis in the body and redistribution of albumin in and out of blood vessels under high inflammatory conditions (41). In patients with liver cirrhosis, although albumin levels are primarily influenced by hepatic synthetic function, it is also influenced by other factors such as decreased protein intake, increase of the catabolic state, increased vascular permeability, systemic inflammatory response, protein-losing enteropathy secondary to portal hypertension, and impaired immunity (38, 40–48). Additionally, Topan et al. reported that low albumin levels were associated with malnutrition in patients with liver cirrhosis (49). Secondly, a previous study has shown that activated and differentiated CD4 + T lymphocytes are recruited to the inflamed liver and cause liver inflammation (50). Lymphopenia has been identified to be a marker of malnutrition and impaired immune response in patients with chronic liver disease (51, 52). Low lymphocytes were found to be associated with mortality in patients with advanced liver cirrhosis who were waiting for liver transplantation (53). Hence, the combination of lymphocytes and albumin primarily reflects inflammatory and immune status and partially reflects malnutrition that may help in predicting the prognosis of patients with DLC.

There are certain drawbacks in the current study. Firstly, it is a single-centered, retrospective, observational study, and selection bias cannot be avoided. Secondly, the predominant etiology of the patients in this study was hepatitis B virus infection, requiring caution to extrapolate these findings to other populations, especially in western cirrhosis populations where alcohol and NASH predominate. Third, this study did not compare the predictive ability of PNI with other nutritional indicators, such as skeletal muscle index or phase angle markers, for the prognosis of patients with DLC. Hence, prospective multicentered research with large sample numbers is required to further evaluate the clinical relevance of the PNI in patients with DLC.

## Conclusion

Prognostic nutritional index may be a potential and promising predictor of prognosis in patients with DLC. It is readily available and could be used to identify patients at high risk of death. This finding may be used to improve the prognosis in patients with DLC by adjusting the treatment strategies in clinical practice.

## Data availability statement

The raw data supporting the conclusions of this article will be made available by the authors, without undue reservation.

## Ethics statement

The studies involving human participants were reviewed and approved by the Scientific Research and New Technology Institutional Review Board of Yijishan Hospital of Wannan Medical College. Written informed consent for participation was not required for this study in accordance with the national legislation and the institutional requirements.

## Author contributions

WW designed the research. YX and CH collected the data. YX and WW analyzed the data and wrote the main manuscript text. YX prepared all tables and figures. WW and CH revised the manuscript. All authors have read and approved the final manuscript.

## Supplementary material

Supplementary Figure 1 The flow chart of the patient selection process.

Supplementary Figure 2 The graphical presentation of the variables on admission. The units of variables were as follows: age, years; white blood cell (WBC), 10^9^/L; lymphocyte (LYM), 10^9^/L; hemoglobin (HGB), g/L; platelet (PLT), 10^9^/L; albumin (ALB), g/L; total bilirubin (TBIL), μmol/L; alanine aminotransferase (ALT), U/L; aspartate aminotransferase (AST), U/L; γ–glutamyl transpeptidase (GGT), U/L; blood urea nitrogen (BUN), mmol/L; creatinine (Cr), μmol/L; prothrombin time (PT), s.

Supplementary Figure 3 Kaplan-Meier analysis curves for survival according to prognostic nutritional index levels in the development and validation cohort.

Supplementary Figure 4 Receiver operating curves of the MELD score, and the prognostic nutritional index for prediction of mortality in patients with decompensated liver cirrhosis in the validation cohort at **(A)** 3, and **(B)** 6 months.
