# Supplementary material for: Prognostic nutritional index: A potential biomarker for predicting the prognosis of decompensated liver cirrhosis
Source: Front Nutr. 2023 Jan 6;9:1092059. doi: 10.3389/fnut.2022.1092059 (PMC9852856; doi:10.3389/fnut.2022.1092059)
Supplement: Supplementary Figure 1 — The flow chart of the patient selection process. [file Data_Sheet_1.PDF]

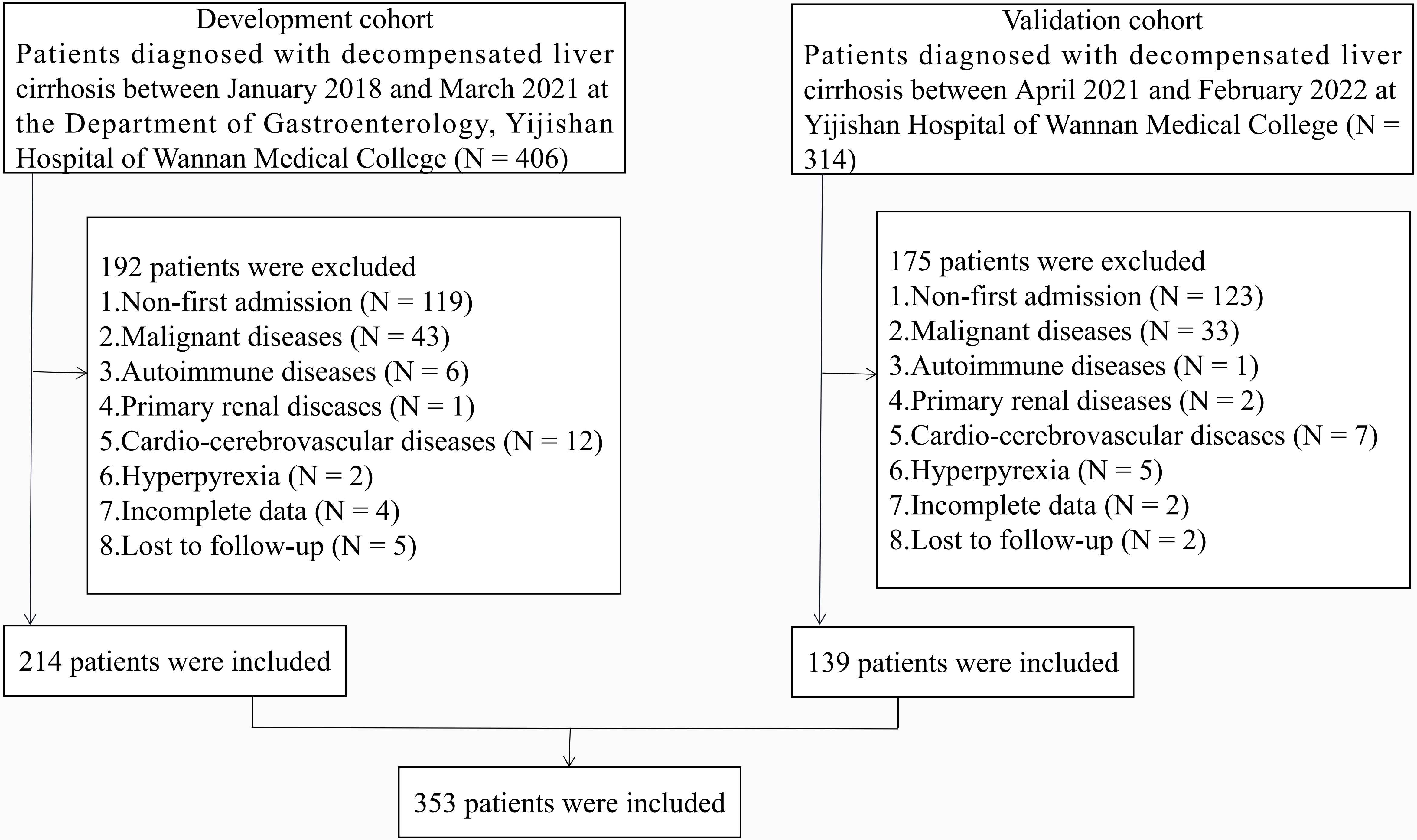
Development cohort
Patients diagnosed with decompensated liver cirrhosis between January 2018 and March 2021 at the Department of Gastroenterology, Yijishan Hospital of Wannan Medical College (N = 406)
192 patients were excluded
1.Non-first admission (N = 119)
2.Malignant diseases (N = 43)
3.Autoimmune diseases (N = 6)
4.Primary renal diseases (N = 1)
5.Cardio-cerebrovascular diseases (N = 12)
6.Hyperpyrexia (N = 2)
7.Incomplete data (N = 4)
8.Lost to follow-up (N = 5)
214 patients were included
Validation cohort
Patients diagnosed with decompensated liver cirrhosis between April 2021 and February 2022 at Yijishan Hospital of Wannan Medical College (N = 314)
175 patients were excluded
1.Non-first admission (N = 123)
2.Malignant diseases (N = 33)
3.Autoimmune diseases (N = 1)
4.Primary renal diseases (N = 2)
5.Cardio-cerebrovascular diseases (N = 7)
6.Hyperpyrexia (N = 5)
7.Incomplete data (N = 2)
8.Lost to follow-up (N = 2)
139 patients were included
353 patients were included
