# Supplementary figures and images for: Prognostic nutritional index: A potential biomarker for predicting the prognosis of decompensated liver cirrhosis
Source: Front Nutr. 2023 Jan 6;9:1092059. doi: 10.3389/fnut.2022.1092059 (PMC9852856; doi:10.3389/fnut.2022.1092059)

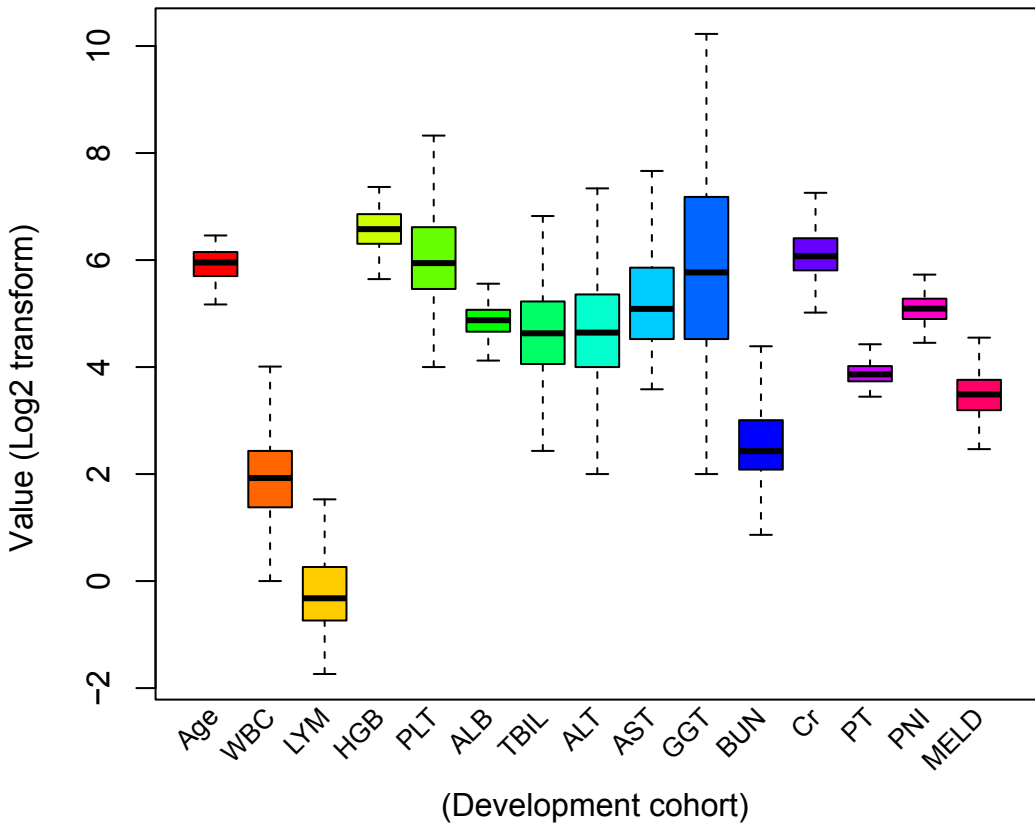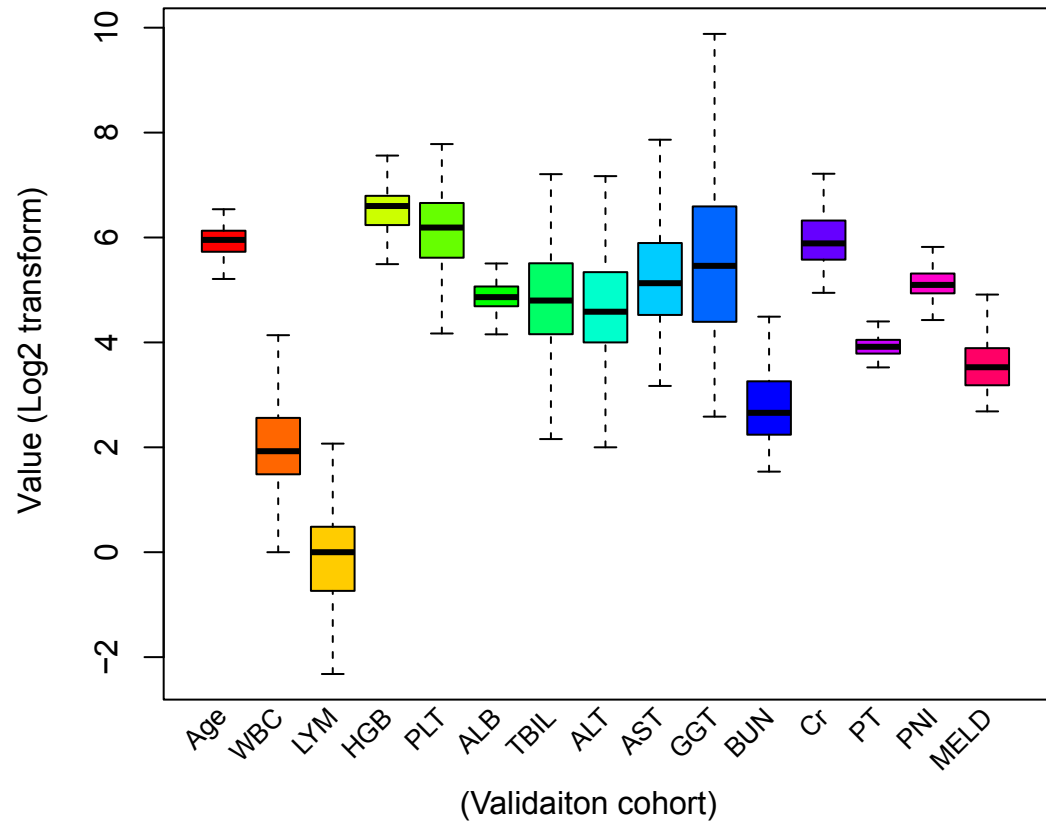

Supplement: Supplementary Figure 2 — The graphical presentation of the variables on admission. The units of variables were as follows: age, years; white blood cell (WBC), 109/L; lymphocyte (LYM), 109/L; hemoglobin (HGB), g/L; platelet (PLT), 109/L; albumin (ALB), g/L; total bilirubin (TBIL), μmol/L; alanine aminotransferase (ALT), U/L; aspartate aminotransferase (AST), U/L; γ–glutamyl transpeptidase (GGT), U/L; blood urea nitrogen (BUN), mmol/L; creatinine (Cr), μmol/L; prothrombin time (PT), s. [file Data_Sheet_2.PDF]

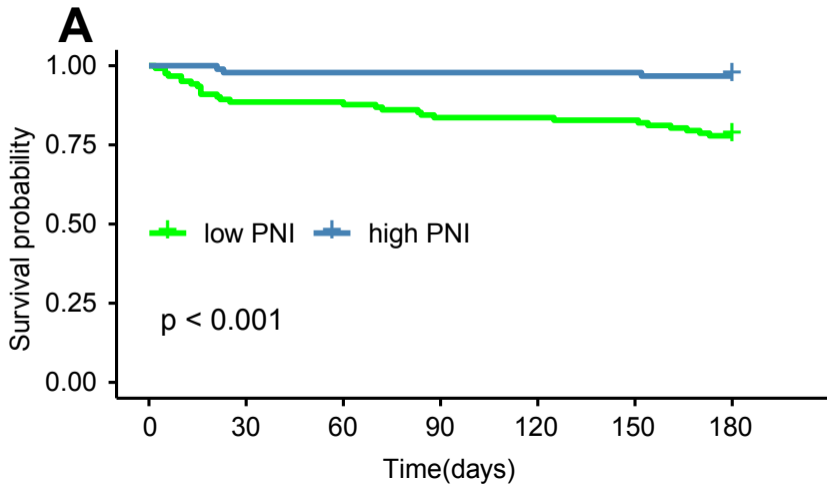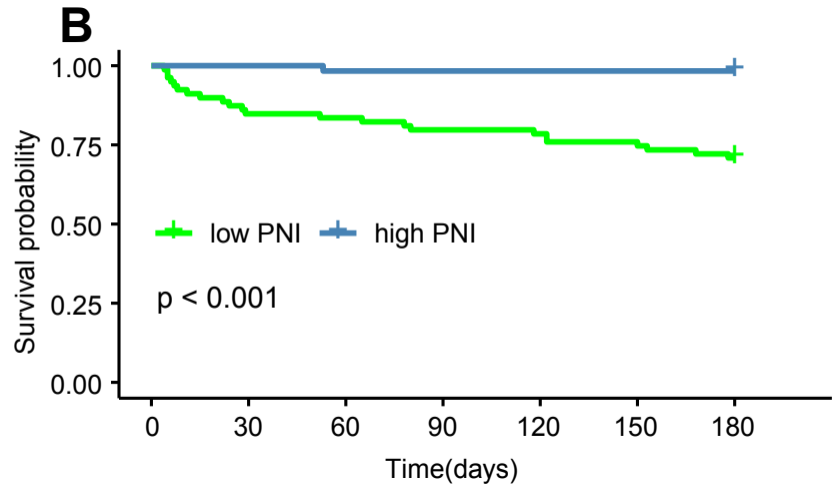

Supplement: Supplementary Figure 3 — Kaplan-Meier analysis curves for survival according to prognostic nutritional index levels in the development and validation cohort. [file Data_Sheet_3.PDF]

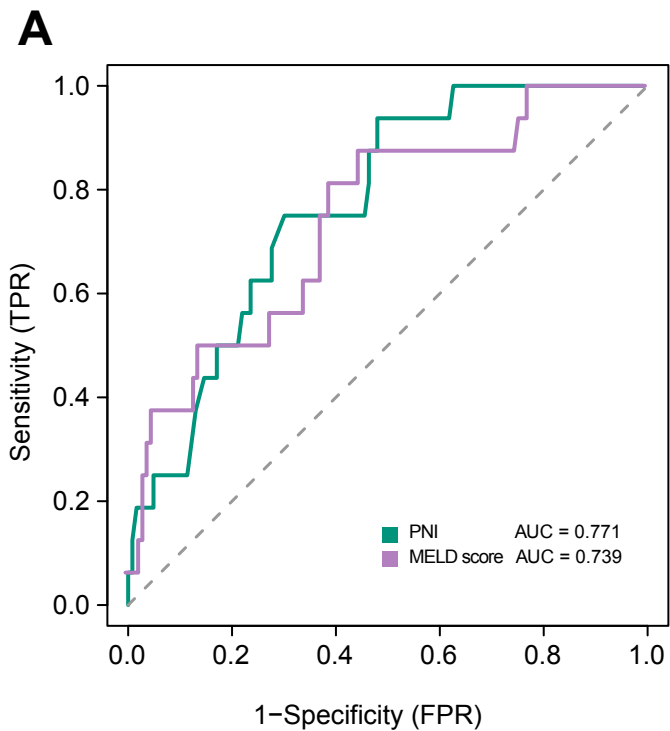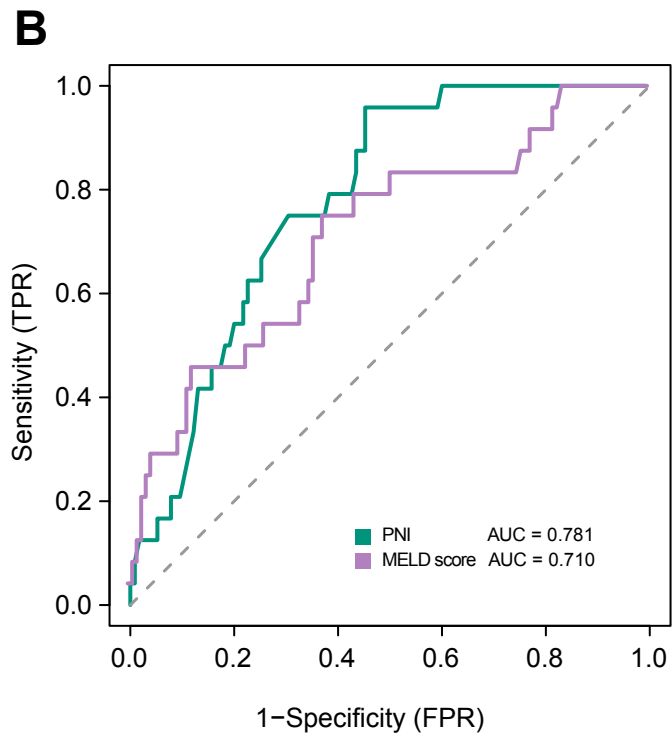

Supplement: Supplementary Figure 4 — Receiver operating curves of the MELD score, and the prognostic nutritional index for prediction of mortality in patients with decompensated liver cirrhosis in the validation cohort at (A) 3, and (B) 6 months. [file Data_Sheet_4.PDF]
